# 1110. Age-related effects of dexmedetomidine, an alpha-2 agonist, on coronary vasoactivity and cardiac function in guinea-pig hearts

**DOI:** 10.1186/2197-425X-2-S1-P89

**Published:** 2014-09-26

**Authors:** M Hongo, S Fujisawa, T Adachi, T Shimbo, S Shibata, T Ohba, K Ono

**Affiliations:** Department of Cell Physiology, Akita University Graduate School of Medicine, Akita City, Japan; Department of Critical Care Medicine, Iwate Medical University, Iwate, Japan

## Introduction

Dexmedetomidine is a potent and selective alpha-2 agonist, and is widely used for not only an adjunct to anesthesia but also sedation during mechanical ventilation in intensive care unit. It has been reported that, although the substance does not appear to have any direct effects on the myocardial contractility, it sometimes causes a decrease in heart rate and a dose-dependent decrease in arterial blood pressure and, in rare cases, leads to cardiac arrest or shock. However, detailed mechanisms remain uncertain. We hypothesized that effects of dexmedetomidine to coronary vasoactivity and cardiac function may differ depending on postnatal ages.

## Objectives

The aim of the present study was to evaluate the effects of dexmedetomidine on cardiac performance and coronary circulation using Langendorff perfused isolated heart of young and adult guinea pigs.

## Methods

Hearts from young (< 4weeks) and adult (>4weeks) guinea-pigs were isolated and mounted on a Langendorff apparatus, and a saline-filled balloon was inserted into the left ventricle. Coronary perfusion pressure (CPP) and the left ventricular pressure (LVP) were continuously monitored and the electro-field stimulation (EFS) was applied to stimulate sympathetic nerve terminals. Also, the effects of dexmedetomidine on the ventricular action potential were evaluated in enzymatically isolated ventricular myocytes.

## Results

Dexmedetomidine almost completely inhibited the increase of LVP induced by EFS with an IC_50_ value of approximately 0.2 nM in both young and adult hearts. On the other hand, the effect on the coronary artery resistance to dexmedetomidine altered during postnatal development, i.e., dexmedetomidine had little effect on CPP in young hearts whereas it increased CPP at concentrations>10 nM in adult hearts (p< 0.05: aged< 4weeks vs. aged 4-8weeks, p< 0.01: aged< 4weeks vs. aged>8weeks). The increase in CPP in adult hearts was inhibited by prazosin, an alpha-1 antagonist (10nM: p=0.0389, 100nM: p=0.0337). Dexmedetomidine had little direct effect on ventricular dP/dt and the action potential of isolated ventricular myocytes.

## Conclusions

The present study confirms the antagonistic action of dexmedetomidine on the increase in ventricular contractility induced by sympathetic stimulation. In addition, the present results demonstrate that the response of coronary artery resistance to dexmedetomidine differs depending on postnatal ages, i.e., dexmedetomidine increases CPP in adult, not in young guinea-pig hearts. The alpha-1 adrenoceptor seems to be involved in the increase of CPP in adult hearts in addition to the alpha-2 adrenoceptor. Aging-associated alteration of the alpha adrenoceptor subtypes might be linked to the cardiodepressant effects of dexmedetomidine.

